# The Correlation of *MGMT* Promoter Methylation and Clinicopathological Features in Gastric Cancer: A Systematic Review and Meta-Analysis

**DOI:** 10.1371/journal.pone.0165509

**Published:** 2016-11-08

**Authors:** Yong Ding, Qihua Yang, Bojun Wang, Guoliang Ye, Xiaoqiong Tong

**Affiliations:** 1 The Affiliated Hospital of Ningbo University, Ningbo, Zhejiang, 315020, People’s Republic of China; 2 The College of Foreign Studies, Ningbo University, Ningbo, Zhejiang, 315211, People’s Republic of China; University of South Alabama Mitchell Cancer Institute, UNITED STATES

## Abstract

The silencing of the tumor suppressor gene O-6-methylguanine-DNA methyltransferase (*MGMT*) by promoter methylation commonly occurs in human cancers. The relationship between *MGMT* promoter methylation and gastric cancer (GC) remains inconsistent. This study aimed to evaluate the potential value of *MGMT* promoter methylation in GC patients. Electronic databases were searched to identify eligible studies. The pooled odds ratio (OR) and the corresponding 95% confidence interval (95% CI) were used to evaluate the effects of *MGMT* methylation on GC risk and clinicopathological characteristics. In total, 31 eligible studies including 2988 GC patients and 2189 nonmalignant controls were involved in meta-analysis. In the pooled analysis, *MGMT* promoter methylation was significantly associated with GC risk (OR = 3.34, P < 0.001) and substantial heterogeneity (P < 0.001). Meta-regression and subgroup analyses based on the testing method, sample material and ethnicity failed to explain the sources of heterogeneity. Interestingly, *MGMT* methylation showed a trend associated with gender, and methylation is lower in males compared with females (OR = 0.76, 95% CI = 0.56–1.03). We did not find a significant association in relation to tumor types, clinical stage, age status or *H*. *pylori* status in cancer (all P > 0.1). *MGMT* promoter methylation may be correlated with the prognosis of GCs in disease free survival (DFS) or overall survival (OS) for univariate analysis. *MGMT* promoter methylation may play a crucial role in the carcinogenesis and prognosis of GC. *MGMT* methylation was not correlated with tumor types, clinical stage, age status, *H*. *pylori* status. However, the result of the association of *MGMT* methylation and gender should be considered with caution.

## Introduction

As one of the most common malignant diseases, gastric cancer (GC) is the third leading cause of cancer-related deaths worldwide. According to global cancer statistics, approximately 951,600 new cases of gastric cancer were diagnosed in 2012, leading to an estimated 723,100 deaths worldwide [1]. *Helicobacter pylori* (*H*. *pylori*) infection affects more than 50% of the adult population in the world and accounts for 75% of all gastric cancer cases [2]. Therefore, *H*. *pylori* infection is a strong risk factor for GC, increasing the risk of developing gastric cancer. GC is divided into two main histological subtypes based on Lauren’s classification: intestinal and diffuse-type gastric cancer [3]. For both types, a strong association with *H*. *pylori*-correlated inflammation exists [4].

Epigenetic alterations are significantly associated with cancer [5]. DNA methylation is a common epigenetic alteration that plays a crucial role in the development of cancer [6, 7]. Accumulative evidence has demonstrated that GC involves a multistep progression process of gastric lesions with complex molecular changes, including DNA methylation [8, 9]. Located on 10q26, O6-methylguanine-DNA-methyltransferase (*MGMT*) encodes a DNA repair protein that counteracts the effect of treatment via removing alkyl adducts from the O6-position of guanine [10]. O6-Alkylated guanine leads to base mismatching and double-strand breaks, thereby inducing apoptosis and cell death [11]. Loss of *MGMT* expression by promoter methylation has been reported in many tumor types [10], including gastric cancer [12]. Therefore, we hypothesized that *MGMT* promoter methylation status might play a role in the development of gastric cancer.

The association between *MGMT* promoter methylation and GC risk remains controversial. Noreikienė et al. reported that the methylation rate of *MGMT* promoter was lower in GC than in non-tumor tissues [13]. Some studies showed that the methylation frequency of *MGMT* promoter was higher in GC than in nonmalignant samples [12, 14]. Therefore, we conducted a meta-analysis to assess the relationship between *MGMT* promoter methylation and GC by comparing cancer cases with nonmalignant controls. Moreover, we also evaluated the correlation between *MGMT* promoter methylation and gender, age status, tumor stage, tumor types and *H*. *pylori* status in cancer.

## Materials and Methods

### Literature search strategy and inclusion criteria

The relevant studies were identified by a systematic search of PubMed, Embase, Cochrane Library and EBSCO databases up to December 25, 2015, without language restrictions. The following key words and search terms were used: (O-6-methylguanine-DNA methyltransferase OR MGMT) AND (stomach OR gastric) AND (cancer OR tumor OR neoplasm OR carcinoma) AND (methylation OR epigene*). Moreover, a manual reference search for relevant articles was also performed to identify the potential additional studies.

Eligible studies had to meet the following inclusion criteria: 1) the study had a diagnosis of primary gastric cancer based on histopathological examination; 2) the study involved *MGMT* promoter methylation frequency in gastric cancer; 3) that study had sufficient data to evaluate the relationship between *MGMT* promoter methylation and gastric cancer; and 4) to avoid duplicated publications, the study selected was the most recent publication or the most complete paper if a series of studies existed. The studies excluded did not meet the inclusion criteria described above.

### Data extraction and quality assessment

The following data were collected for eligible studies: the first author’s name, year of publication, country of origin, ethnicity, sample types, testing method, the number of gastric cancer patients, the number of control group, the number of methylation positive, expression information, clinicopathological parameters (i.e., tumor stage, tumor histotype, age status, sex status and *Helicobacter pylori* (*H*. *pylori*) infection status. Tumor stages 1–2 were defined as early stage, and tumor stages 3–4 were defined as advanced stage. Our study was reported based on the Preferred Reporting Items for Systematic Reviews and Meta-Analysis (PRISMA) statement (S1 Table). Moreover, two reviewers independently estimated the quality of eligible studies according to Newcastle–Ottawa Scale (NOS) for case–control or cohort studies [15, 16], including three parameters of quality: selection (0–4), comparability (0–2), and outcome or exposure assessment (0–3). In this study, NOS scores ranged from 0 to 9 for each study, the study with 6 or more scores was considered as high quality, and a NOS score of less than 6 was considered as low quality [15].

### Statistical analysis

Stata software (version 12.0, Stata Corporation, College Station, TX, USA) was used for statistical analysis. The overall odds ratio (OR) and the corresponding 95% confidence interval (95% CI) were calculated to evaluate the association between *MGMT* promoter methylation and GC risk. In addition, the association of *MGMT* promoter methylation and clinicopathological features was also assessed via the pooled OR with 95% CI. Statistical heterogeneity was examined using the chi-square test and Q statistics [17]. If heterogeneity was significant (I^2^ ≥ 50% or p < 0.1), the random-effects model was used. Meta-regression analyses and subgroup analyses were performed to further evaluate the sources of heterogeneity. Otherwise, a fixed-effects model was used [18, 19]. A sensitivity analysis was also conducted to assess the influence and stability of an individual study on the pooled OR by deleting one study [20]. The publication bias was detected using Egger’s test for the analysis with greater 9 studies [21]. We also conducted a cumulative meta-analysis by precision method to evaluate the possible publication bias for the result with less than 10 studies [22].

## Results

### Study characteristics

**Fig 1:**
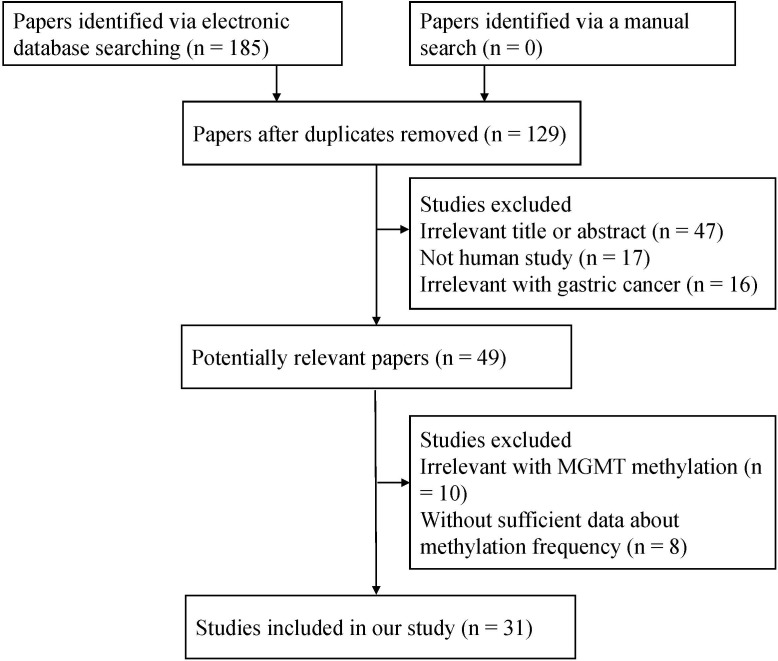
Flow chart of the literature search strategy.

Initially, a total of 185 studies were identified by searching electronic databases. Based on the inclusion criteria described above, 31 studies [12–14, 23–49] [50] that reported the sufficient data were ultimately included in the current meta-analysis (Fig 1), including a total of 2988 GC patients and 2189 nonmalignant controls. Of these studies, 20 studies reporting 2120 cases and 2189 nonmalignant controls were calculated to assess the association between *MGMT* methylation and GC risk, and 17 studies reporting 1299 male GC patients and 775 female GC patients were used to evaluate the association between *MGMT* methylation and gender. Furthermore, 11 studies, including 464 patients with intestinal gastric cancer and 416 patients with diffuse gastric cancer, evaluated the association between *MGMT* methylation and tumor type; 10 studies including 221 stage 1–2 patients and 469 stage 3–4 patients evaluated the association between *MGMT* methylation and tumor stage; 9 studies assessed the correlation between *MGMT* promoter methylation and age status (more than or equal to 60 years: 387 GC patients, less than or equal to 60 years: 315 GC patients); and 3 studies involving 139 *H*. *pylori*-positive patients and 147 *H*. *pylori*-negative patients explored the association between *MGMT* methylation and *H*. *pylori* infection status. 2 studies with 198 GC patients reported survival. The basic characteristics of included studies were presented in S2 Table.

### *MGMT* gene methylation and risk of GC

**Fig 2:**
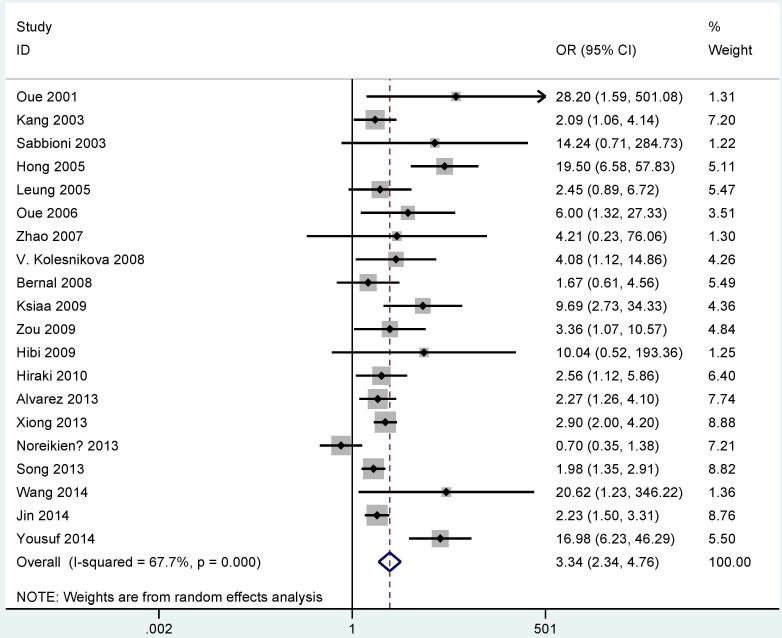
Forest plot of the correlation between *MGMT* methylation and GC.

In the comparison of GC and control groups, substantial heterogeneity was obvious (I^2^ = 67.7% and P < 0.001); thus, a random-effects model was used. The result showed that the overall OR for *MGMT* promoter methylation in cancer cases compared with nonmalignant controls was 3.34 (95% CI = 2.34–4.76, P < 0.001) (Fig 2).

### Subgroup analyses of *MGMT* promoter methylation

The subgroup analyses were conducted based on the methylation detection method (MSP, MethyLight or Pyrosequencing), sample material (fresh frozen tissue, formalin-fixed paraffin-embedded tissue or blood) and race (Caucasians, Asians or mixed population) (Table 1). In the subgroup analysis of the testing method, the pooled OR was 3.56 (95% CI = 2.30–5.51, P < 0.001) for the MSP subgroup among 15 studies, 3.62 (95% CI = 2.01–6.53, P < 0.001) for the MethyLight subgroup among 4 studies, and 2.27 (95% CI = 1.26–4.10, P = 0.006) for the Pyrosequencing subgroup in 1 study. In the subgroup analysis of the sample material, the OR value for the fresh frozen (FF) tissue subgroup was 3.86 (95% CI = 2.24–6.63, P < 0.001) among 11 studies. The OR for the formalin-fixed paraffin-embedded (FFPE) tissue subgroup was 2.68 (95% CI = 1.87–3.82, P < 0.001) among 6 studies, and the OR for the blood sample subgroup was 2.97 (95% CI = 1.35–6.57, P = 0.007) among 2 studies. The result by subgroup analysis of race revealed that *MGMT* methylation was significantly associated with GC risk in Asian and Caucasian populations (OR = 3.80, 95% CI = 2.56–5.64, P < 0.001; OR = 2.91, 95% CI = 1.07–7.89, P = 0.036; respectively) among 14 studies and 5 studies, respectively, but not in the mixed population in one study (P = 0.316).

**Table 1:** The summary of OR in cancer vs. control.

	Studies	Overall OR (95 CI %)	I^2^; p	P-value	Cases	Controls	p (Egger's test)
Total	20	3.34 (2.34–4.76)	67.7; < 0.001	< 0.001	2120	2189	0.021
Subgroup							
Method							
MSP	15	3.56 (2.30–5.51)	74.8%; < 0.001	< 0.001	1747	1937	0.063
MethyLight	4	3.62 (2.01–6.53)	5.2%; 0.367	< 0.001	281	155	NA
PSQ	1	2.27 (1.26–4.10)	NA; NA	0.006	92	97	NA
Material							
FFT	11	3.86 (2.24–6.63)	80.5%; < 0.001	< 0.001	1615	1645	0.087
FFPE	6	2.68 (1.87–3.82)	14.6%; 0.320	< 0.001	404	494	NA
Blood	2	2.97 (1.35–6.57)	0.0%; 0.541	0.007	80	44	NA
Race							
Caucasians	5	2.91 (1.07–7.89)	78.2%; 0.001	0.036	270	247	NA
Mix	1	1.67 (0.61–4.56)	NA; NA	0.316	47	47	NA
Asians	14	3.80 (2.56–5.64)	64.8%; < 0.001	< 0.001	1803	1895	0.016

Mix: mixed population; PSQ: Pyrosequencing; FFT: fresh frozen tissue; FFPE: formalin-fixed and paraffin-embedded tissue; MSP: methylation-specific polymerase chain reaction; NA: not applicable; OR: odds ratio; 95% CI: 95% confidence interval.

### Meta-regression and subgroup analyses in the GC and control group

According to the methylation detection method (MSP, MethyLight or Pyrosequencing), sample material (fresh frozen tissue, formalin-fixed paraffin-embedded tissue or blood) and race (Caucasians, Asians or mixed population), subgroup analysis (Table 1) and meta-regression analysis (Table 2) were performed to explore the potential sources of heterogeneity. Heterogeneity based on subgroup analysis of the detection method revealed significant differences (MSP subgroup: I^2^ = 74.8%, P < 0.001; MethyLight subgroup: I^2^ = 5.2%, P = 0.367). Significantly different evidence of heterogeneity was noted in different sample material subgroups (FF tissue subgroup: I^2^ = 80.5%, P < 0.001; FFPE tissue subgroup: I^2^ = 14.6%, P = 0.320; blood sample subgroup: I^2^ = 0.0%, P = 0.541). Heterogeneity was observed within different ethnicity subgroups (Caucasian population subgroup: I^2^ = 78.2%, P = 0.001; Asian population subgroup: I^2^ = 64.8%, P < 0.001). The result revealed that subgroup analyses did not identify the sources of heterogeneity.

**Table 2:** Meta-regression analysis in cancer vs. control.

Subgroup	Coefficient (95% CI)	t	P value
Sample material			0.81
FFPE	-0.056 (-1.791, 1.680)	-0.07	0.947
FFT	0.263 (-1.374, 1.899)	0.34	0.738
Ethnicity			0.51
Asians	0.919 (-1.108, 2.945)	0.96	0.352
Caucasians	0.496 (-1.662, 2.653)	0.48	0.634
Testing method			0.857
PSQ	-0.516 (-2.715, 1.684)	-0.49	0.627
MSP	-0.018 (-1.358, 1.323)	-0.03	0.978

PSQ: Pyrosequencing; FFT: fresh frozen tissue; FFPE: formalin-fixed and paraffin-embedded tissue; MSP: methylation-specific polymerase chain reaction; 95% CI: 95% confidence interval.

The following meta-regression analysis was used. However, the result of meta-regression analysis showed that the methylation detection method, sample material and ethnicity failed to identify the source of heterogeneity (P > 0.1). This result was consistent with the subgroup analysis.

### The association between *MGMT* methylation and clinicopathological features

**Fig 3:**
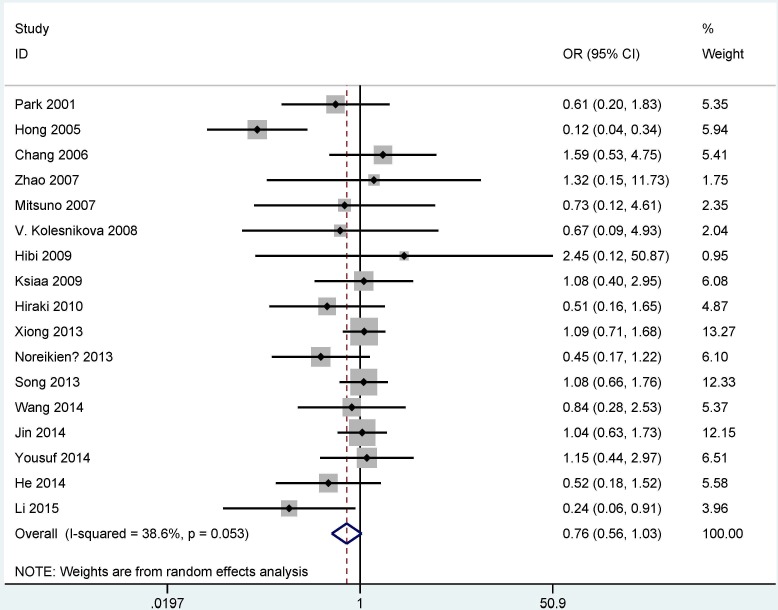
Forest plot of the correlation between *MGMT* methylation and gender.

Table 3 showed the relationship between *MGMT* methylation and clinicopathological features. The analyses of the correlation of *MGMT* methylation, gender, tumor types, and tumor stage used the random-effects model (all P < 0.1), but a fixed-effects model was used for *H*. *pylori* infection status and age status (P > 0.1). The result suggested that *MGMT* methylation had a trend toward less frequency in male gastric cancer patients compared with female gastric cancer patients (OR = 0.76, 95% CI = 0.56–1.03, P = 0.077) (Fig 3). No significant differences in *MGMT* methylation were noted in relation to tumor type, tumor stage, age status and *H*. *pylori* infection status in GC (all P > 0.1) (Table 3).

**Table 3:** The correlation of *MGMT* promoter methylation and clinicopathological features.

	Studies	Overall OR (95CI %)	I^2^; p	P value	GC patients		p (Egger's test)
Gender					Male	Female	
	17	0.76 (0.56–1.03)	38.6%; 0.053	0.077	1299	775	0.167
Tumor stage					Stage 1–2	Stage 3–4	
	10	0.65 (0.33–1.26)	46.4%; 0.052	0.205	221	469	0.97
Tumor types					Intestinal	Diffuse	
	11	1.09 (0.66–1.78)	50.3%; 0.028	0.74	464	416	0.105
Age					>/ = 60 years	</ = 60 years	
	9	1.03 (0.71–1.49)	8.9%; 0.361	0.897	387	315	NA
H. pylori					Positive	Negative	
	3	1.19 (0.66–2.15)	0.0%; 0.823	0.564	139	147	NA

NA: not applicable; OR: odds ratio; 95% CI: 95% confidence interval.

### *MGMT* promoter methylation as a prognostic factor for GC

Two studies reported the prognosis of *MGMT* promoter methylation [48, 50]. Park et al. reported that there was significant association between *MGMT* promoter methylation and 5-year disease free survival (DFS) for univariate analysis (P < 0.02) [48]. Shi et al. reported that *MGMT* promoter methylation was correlated with overall survival (OS) of GCs using univariate analysis [50].

### Sensitivity analysis

To assess the stability of the overall OR and the change of heterogeneity based on the omission of single study, sensitivity analyses were conducted in cancer cases vs. nonmalignant controls and male cancer cases vs. female cancer cases. In the comparison of cancer cases and controls, when Noreikienė 2013 et al. ([13], Lithuania), Yousuf 2014 et al. ([12], China) and Hong 2005 et al. ([43], Korea) were successively removed, heterogeneity was significantly decreased (P-values were 0.001, 0.021 and 0.364, respectively); however, the pooled OR was not significantly changed (ORs were 3.62, 3.11 and 2.58, respectively). The overall OR between *MGMT* methylation and gender in cancer was substantially changed based on omission of Hong 2005 et al. ([43], Korea), with a change from 0.76 (95% CI = 0.56–1.03) to 0.93 (95% CI = 0.75–1.15) and no heterogeneity (P = 0.704).

### Publication bias

As shown in Tables 1 and 3, slight publication bias was detected by Egger’s test only in the comparison of cancer samples and control samples and in the Asian population subgroup (P = 0.021 and P = 0.016, respectively). When cancer was compared to controls, we removed two studies with low quality [34, 44], and re-calculated the pooled OR (OR = 3.26, 95% CI = 2.26–4.71, P < 0.001), with a slight publication bias (P = 0.039). Obvious publication bias was not noted in other analyses for the result with more than 9 studies (all P > 0.05). For the analysis with less than 10 studies, a cumulative meta-analysis by precision method did not find obvious evidence of publication bias (S3 Table).

## Discussion

The hypermethylation of tumor suppressor genes and hypomethylation of oncogenes are two essential molecular mechanisms of epigenomic regulation, which play key roles in the initiation and progression of cancer [51–53]. *MGMT* has been reported as a tumor suppressor gene in colorectal cancer [54]. The methylation status of the *MGMT* promoter has been observed in some cancers, such as non-small cell lung cancer [55], glioblastoma [56], and breast cancer [57]. Several studies showed that significant association was found between *MGMT* promoter methylation and its expression in GC, with loss of *MGMT* expression [12, 32, 40, 49]. In addition, the methylation frequency of the *MGMT* promoter was inconsistent in gastric cancer, with a range from 7% [38] to 70% [34]. Noreikienė et al. reported that the methylation level of *MGMT* promoter was 36.2% in GC samples, and 44.9% in non-tumor tissues [13]. Some studies reported that *MGMT* promoter methylation frequency was higher in GC than in non-tumor samples [12, 14, 33]. Therefore, we performed a meta-analysis to evaluate the correlation between *MGMT* promoter methylation and GC. In the current study, the methylation frequency of *MGMT* promoter was inconsistent in GC, subgroup analysis of DNA methylation testing method revealed that *MGMT* promoter methylation had a similar frequency in different methods. Thus, the possible reason of inconsistent methylation frequency of the *MGMT* may be different CpG sites of the promoter.

Our findings showed that the *MGMT* methylation status was significantly associated with the risk of GC (OR = 3.34, 95% CI = 2.34–4.76, P < 0.001), suggesting that *MGMT* methylation can be crucial for the carcinogenesis of gastric cancer.

Further subgroup analyses were conducted according to the methylation detection method (MSP, MethyLight or Pyrosequencing), sample material (fresh frozen tissue, formalin-fixed paraffin-embedded tissue or blood) and race (Caucasians, Asians or mixed population). The results showed that the association between *MGMT* methylation and GC was correlated with different detection methods and different sample materials. Subgroup analysis based on ethnicity demonstrated that *MGMT* methylation was significantly associated with GC in the Asian (OR = 3.80, P < 0.001) and Caucasian populations (OR = 2.91, P = 0.036) but not in a mixed population (P = 0.316). However, the results should be carefully considered as only one study or two studies with small sample sizes were included in the Pyrosequencing, blood sample, and mixed population subgroups.

Significant heterogeneity existed in cancer cases compared with controls (P < 0.001). Therefore, we performed meta-regression and subgroup analyses to explain the sources of heterogeneity. The results of subgroup analyses and meta-regression analyses were consistent but were unable to identify the sources of heterogeneity. The following sensitivity analysis was conducted to identify the stability of the overall OR by deleting individual studies. Three studies (Noreikienė 2013 et al., Yousuf 2014 et al. and Hong 2005 et al.) were successively removed, and the pooled OR (OR = 2.58, 95% CI = 2.12–3.14, P < 0.001) remained significant with no evidence of heterogeneity (P = 0.364). However, the value was slightly smaller than that in the current meta-analysis (OR = 3.34, 95% CI = 2.34–4.76, P < 0.001), suggesting that a significant association existed between *MGMT* methylation and GC. Therefore, our result was stable and reliable.

We further analyzed the clinicopathological significance of *MGMT* promoter methylation in GC patients. For gender status, the overall OR was 0.76 (95% CI = 0.56–1.03) in 1299 male GC patients and 775 female GC patients, indicating that the *MGMT* methylation status had a trend associated with gender status. The result showed that methylated *MGMT* may be a susceptible gene for female GC patients. Based on the existence of heterogeneity (I^2^ = 38.6% and P = 0.053), the result of sensitivity analysis by omitting a single study (Hong 2005 et al.: 64 male patients and 36 female patients) showed that the summary OR was 0.93 (95% CI = 0.75–1.15), suggesting that *MGMT* methylation was not correlated with gender status, with no evidence of heterogeneity (P = 0.704). This result should be applied with caution. In addition, only two studies with small sample sizes (136 male GC patients and 66 female GC patients) reported that *MGMT* promoter methylation rate was significantly lower in male than in female [25, 43]. Although the present study was shown to be methylated in the promoter, the included studies did not state specific location of CpG sites of the *MGMT* promoter. Therefore, the above analysis of *MGMT* promoter methylation with gender status may be still required to confirm the result in detail in the future. Other clinicopathological features were also analyzed, including tumor stage (OR = 0.65, 95% CI = 0.33–1.26), tumor type (OR = 1.09, 95% CI = 0.66–1.78), age status (OR = 1.03, 95% CI = 0.71–1.49), and *H*. *pylori* infection status (OR = 1.19, 95% CI = 0.66–2.15). The results suggested that *MGMT* methylation was not associated with tumor stage, tumor type, age status or *H*. *pylori* infection status.

When GC was compared to nonmalignant specimens, a slight publication bias was observed (P = 0.021). We determined whether these studies excluded with low quality contributed to reduce the potential publication bias. When two studies were deleted [34, 44], we found that the combined OR was not significantly changed (OR = 3.26, P < 0.001), a slight publication bias was also detected in the remaining 18 studies (P = 0.039), which suggested that poor-quality studies did not mainly impact the risk of bias. In addition, we deleted two studies with high quality [31, 47], no evidence of publication bias was observed in the remaining 18 studies (P = 0.081 > 0.05), indicating the stability of our analyses. For the result with fewer than 10 studies, a cumulative meta-analysis was analyzed in our study. The result showed that no significant publication bias was found in relation to age status and *H*. *pylori* infection status etc. (n < 10). Based on the smaller studies or sample sizes, further well-designed, large-scale studies are very essential to validate our results in the future.

This study had several limitations. First, the PubMed, Embase, Cochrane Library and EBSCO databases were used to minimize publication bias. However, publication bias was detected based on Egger’s test in cancer case vs. controls (P = 0.021) and in the Asian population subgroup (P = 0.016). The papers with positive results are more often published than papers with negative results. Articles with other styles, such as unpublished studies and conference abstracts, were excluded due to insufficient data. Second, the main ethnic populations were Asians and Caucasians, and other ethnicities, such as Africans, were limited. Therefore, the association between *MGMT* methylation and other ethnicities was not evaluated based on insufficient data. Third, the sample size of some subgroup analyses, such as blood sample and mixed population, were smaller. Fourth, one study with 79 patients reported that *MGMT* promoter methylation was notably correlated with 5-year disease free survival (DFS) in univariate analysis. One study with 119 patients reported that significant correlation was found between *MGMT* promoter methylation and OS for univariate analysis. These results should be carefully considered, and more studies with large sample size should be performed in the future.

In conclusion, the results showed that *MGMT* methylation may play a key role in GC initiation. It may be correlated with DFS and OS of GC patients in univariate analysis. In addition, we did not find that *MGMT* promoter methylation was associated with tumor histology, tumor stage, age status, *H*. *pylori* status in GC patients. The result of the correlation between *MGMT* methylation and gender was not stable, which should be conservatively considered.

## Supporting Information

S1 File Checklists for meta-analysis on genetic association studies. (DOCX)

S1 Table PRISMA 2009 Checklist. (DOC)

S2 Table The basic characteristics of eligible studies. (DOC)

S3 Table Cumulative forest plot of publication bias of *MGMT* promoter methylation for studies with less than 10 studies. (DOC)
