# Supplementary material for: The Correlation of MGMT Promoter Methylation and Clinicopathological Features in Gastric Cancer: A Systematic Review and Meta-Analysis
Source: PLoS One. 2016 Nov 8;11(11):e0165509. doi: 10.1371/journal.pone.0165509 (PMC5100908; doi:10.1371/journal.pone.0165509)
Supplement: S2 Table — (DOC) [file pone.0165509.s003.doc]

Table S2: The basic characteristics of eligible studies.

| First author | Country | Ethnicity | Mean year | Method | Sample | Case | Control | >/=60 years | </=60 years | Male | Female | Stage 1-2 | Stage 3-4 | Intestinal | Diffuse | *H. pylori +* | *H. pylori -* | Survival | Expression | NOS |
| --- | --- | --- | --- | --- | --- | --- | --- | --- | --- | --- | --- | --- | --- | --- | --- | --- | --- | --- | --- | --- |
|  |  |  |  |  |  | M+/N | M+/N | M+/N | M+/N | M+/N | M+/N | M+/N | M+/N | M+/N | M+/N | M+/N | M+/N |  |  |  |
| Oue 2001 [49] | Japan | Asians | Not clear | MSP | FFT | 8/26 |  |  |  |  |  | 5/10 | 3/16 | 7/16 | 1/10 |  |  | No | Negative | 6 |
| Oue 2001 [47] | Japan | Asians | Not clear | MSP | FFT | 8/50 | 0/70 |  |  |  |  |  |  | 7/24 | 1/26 |  |  | No | ND | 6 |
| Park 2001 [48] | Korea | Asians | 56.4 | MSP | FFT | 18/79 |  |  |  | 11/55 | 7/24 | 3/29 | 15/50 |  |  |  |  | Yes | NS | 8 |
| Kang 2003 [46] | Korea | Asians | Not clear | MSP | FFPE | 17/80 | 24/210 |  |  |  |  |  |  |  |  |  |  | No | ND | 6 |
| Oue 2003 [45] | Japan | Asians | Not clear | MSP | FFT | 26/103 |  |  |  |  |  |  |  | 19/51 | 7/52 |  |  | No | ND | 7 |
| Sabbioni 2003 [44] | Italy | Caucasians | Not clear | MSP | Tissue | 11/21 | 0/6 |  |  |  |  |  |  |  |  |  |  | No | ND | 5 |
| Hong 2005 [43] | Korea | Asians | Not clear | MSP | FFT | 25/100 | 4/238 | 15/63 | 10/37 | 7/64 | 18/36 |  |  | 8/30 | 13/45 | 17/63 | 8/37 | No | ND | 8 |
| Leung 2005 [42] | China | Asians | 66 | * | Blood | 35/60 | 8/22 |  |  |  |  |  |  |  |  |  |  | No | ND | 9 |
| Motoshita 2005 [41] | Japan | Asians | Not clear | MSP | FFT | 11/33 |  | 7/27 | 4/6 |  |  |  |  |  |  |  |  | No | ND | 6 |
| Oue 2006 [40] | Japan | Asians | 68.6 | MSP | FFT | 20/75 | 2/35 |  |  |  |  |  |  |  |  |  |  | No | Negative | 7 |
| Chang 2006 [39] | Japan | Asians | Not clear | MSP | FFT | 27/106 |  | 19/70 | 8/36 | 22/80 | 5/26 |  |  | 17/62 | 10/44 |  |  | No | ND | 8 |
| Cai 2007 [36] | China | Asians | 57.6 | MSP | FFPE | 19/106 |  |  |  |  |  | 0/17 | 19/89 | 7/44 | 12/62 |  |  | No | ND | 6 |
| Zhao 2007 [38] | China | Asians | Not clear | MSP | FFPE | 7/101 | 0/26 |  |  | 6/83 | 1/18 | 3/27 | 4/73 |  |  | 2/35 | 5/65 | No | ND | 6 |
| Mitsuno 2007 [37] | Japan | Asians | 67.3 | MSP | FFT | 7/38 |  |  |  | 5/29 | 2/9 | 0/4 | 7/34 |  |  |  |  | No | ND | 8 |
| Kolesnikova 2008 [34] | Russia | Caucasians | Not clear | MSP | Blood | 14/20 | 8/22 | 6/6 | 8/14 | 8/12 | 6/8 |  |  |  |  |  |  | No | ND | 5 |
| Bernal 2008 [35] | Chile | Mix | Not clear | MSP | FFPE | 12/47 | 8/47 |  |  |  |  |  |  |  |  |  |  | No | ND | 7 |
| Ksiaa 2009 [33] | Tunisia | Caucasians | 61.1 | MSP | FFPE | 25/68 | 3/53 | 20/54 | 5/14 | 15/40 | 10/28 |  |  |  |  |  |  | No | ND | 7 |
| Zou 2009 [32] | China | Asians | Not clear | MSP | FFPE | 8/16 | 14/61 |  |  |  |  |  |  |  |  |  |  | No | Negative | 6 |
| Hibi 2009 [30] | Japan | Asians | Not clear | * | FFT | 4/38 | 0/38 |  |  | 4/31 | 0/7 | 0/15 | 4/23 |  |  |  |  | No | ND | 8 |
| Hiraki 2010 [31] | Japan | Asians | 68.6 | * | FFT | 26/49 | 15/49 |  |  | 14/30 | 12/19 | 11/24 | 15/25 | 13/27 | 13/22 |  |  | No | ND | 8 |
| Schneider 2010 [29] | USA | Caucasians | Not clear | PSQ | FFPE | 48/86 |  |  |  |  |  |  |  |  |  | 24/41 | 24/45 | No | ND | 7 |
| Shi 2012 [50] | China | Asians | 59.2 | MSP | FFPE | 10/119 |  |  |  |  |  |  |  |  |  |  |  | Yes | ND | 8 |
| Alvarez 2013 [28] | Brazil | Caucasians | 60 | PSQ | FFPE | 61/92 | 45/97 |  |  |  |  |  |  |  |  |  |  | No | NG | 7 |
| Xiong 2013 [27] | China | Asians | 52.8 | MSP | FFT | 114/413 | 48/413 |  |  | 63/222 | 51/191 |  |  |  |  |  |  | No | ND | 7 |
| Noreikienė 2013 [13] | Lithuania | Caucasians | 64.5 | MSP | FFT | 25/69 | 31/69 | 17/48 | 8/21 | 11/39 | 14/30 |  |  | 10/35 | 14/32 |  |  | No | ND | 8 |
| Song 2013 [26] | China | Asians | 54.5 | MSP | FFT | 89/322 | 52/322 |  |  | 49/173 | 40/149 |  |  |  |  |  |  | No | ND | 7 |
| Wang 2014 [14] | China | Asians | 59 | * | FFT | 24/134 | 0/46 | 14/64 | 10/70 | 19/109 | 5/25 |  |  | 7/52 | 13/67 |  |  | No | ND | 6 |
| Jin 2014 [24] | China | Asians | 53.7 | MSP | FFT | 90/283 | 49/283 |  |  | 49/152 | 41/131 |  |  |  |  |  |  | No | ND | 7 |
| Yousuf 2014 [12] | China | Asians | Not clear | MSP | FFT | 43/82 | 5/82 |  |  | 31/58 | 12/24 | 16/43 | 27/39 | 28/59 | 15/23 |  |  | No | Negative | 8 |
| He 2014 [23] | China | Asians | Not clear | MSP | Tissue | 24/70 |  | 9/26 | 15/44 | 15/50 | 9/20 | 12/33 | 12/37 |  |  |  |  | No | ND | 7 |
| Li 2015 [25] | China | Asians | 53 | nMSP | FFPE | 10/102 |  | 1/29 | 9/73 | 4/72 | 6/30 | 2/19 | 8/83 | 7/64 | 3/33 |  |  | No | ND | 8 |

Mix: mixed population; “*” stands for MethyLight; PSQ: Pyrosequencing; H. pylori: Helicobacter pylori; FFT: fresh frozen tissue; FFPE: formalin-fixed and paraffin-embedded tissue; nMSP: nested methylation-specific polymerase chain reaction; MSP: methylation-specific polymerase chain reaction; M+: the number of methylation; N: the number of methylation and unmethylation; ND: not done; NOS: Newcastle–Ottawa Scale; NS: No significant association.
