# Supplementary material for: The Correlation of MGMT Promoter Methylation and Clinicopathological Features in Gastric Cancer: A Systematic Review and Meta-Analysis
Source: PLoS One. 2016 Nov 8;11(11):e0165509. doi: 10.1371/journal.pone.0165509 (PMC5100908; doi:10.1371/journal.pone.0165509)
Supplement: S3 Table — (DOC) [file pone.0165509.s004.doc]

Cumulative forest plot of publication bias of *MGMT* promoter methylation for the analyses with less than 10 studies·

Cumulative forest plot of publication bias from 9 studies in relation to age status in cancer

Cumulative forest plot of publication bias from 5 studies between *MGMT* promoter methylation and Caucasian population with gastric cancer

Cumulative forest plot of publication bias from 6 studies between *MGMT* promoter methylation and gastric cancer in formalin-fixed and paraffin-embedded tissue (FFPE) subgroup

Cumulative forest plot of publication bias from 4 studies between *MGMT* promoter methylation and gastric cancer in MethyLight subgroup

Cumulative forest plot of publication bias from 3 studies in relation to *Helicobacter pylori* (*H. pylori*) status in cancer
